# Hsp70: A Multifunctional Chaperone in Maintaining Proteostasis and Its Implications in Human Disease

**DOI:** 10.3390/cells14070509

**Published:** 2025-03-29

**Authors:** Manish Kumar Singh, Sunhee Han, Songhyun Ju, Jyotsna S. Ranbhise, Joohun Ha, Seung Geun Yeo, Sung Soo Kim, Insug Kang

**Affiliations:** 1 Department of Biochemistry and Molecular Biology, School of Medicine, Kyung Hee University, Seoul 02447, Republic of Korea; manishbiochem@gmail.com (M.K.S.); sunheehan@khu.ac.kr (S.H.); thdgus8543@khu.ac.kr (S.J.); jogm25@khu.ac.kr (J.S.R.); hajh@khu.ac.kr (J.H.); 2 Biomedical Science Institute, Kyung Hee University, Seoul 02447, Republic of Korea; 3 Department of Biomedical Science, Graduate School, Kyung Hee University, Seoul 02447, Republic of Korea; 4 Department of Otorhinolaryngology—Head and Neck Surgery, College of Medicine, Kyung Hee University Medical Center, Kyung Hee University, Seoul 02453, Republic of Korea; yeo2park@gmail.com

**Keywords:** cancer, epichaperome, Hsp70, inflammation, apoptosis, protein-folding

## Abstract

Hsp70, a 70 kDa molecular chaperone, plays a crucial role in maintaining protein homeostasis. It interacts with the DnaJ family of co-chaperones to modulate the functions of client proteins involved in various cellular processes, including transmembrane transport, extracellular vesicle trafficking, complex formation, and proteasomal degradation. Its presence in multiple cellular organelles enables it to mediate stress responses, apoptosis, and inflammation, highlighting its significance in disease progression. Initially recognized for its essential roles in protein folding, disaggregation, and degradation, later studies have demonstrated its involvement in several human diseases. Notably, Hsp70 is upregulated in multiple cancers, where it promotes tumor proliferation and serves as a tumor immunogen. Additionally, epichaperome networks stabilize protein–protein interactions in large and long-lived assemblies, contributing to both cancer progression and neurodegeneration. However, extracellular Hsp70 (eHsp70) in the tumor microenvironment can activate immune cells, such as natural killer (NK) cells, suggesting its potential in immunotherapeutic interventions, including CAR T-cell therapy. Given its multifaceted roles in cellular physiology and pathology, Hsp70 holds immense potential as both a biomarker and a therapeutic target across multiple human diseases. This review highlights the structural and functional importance of Hsp70, explores its role in disease pathogenesis, and discusses its potential in diagnostic and therapeutic applications.

## 1. Introduction

Stress is a universal phenomenon experienced by all organisms, manifesting in both acute and chronic forms. It impacts cellular physiology and disrupts protein homeostasis. Prolonged exposure to stresses results in various pathological conditions, contributing to a diverse range of diseases due to its detrimental effects on tissue injury and repair, including stroke, cancer, neurodegeneration, cardiac disorders, and atherosclerosis [1]. In response to stress, cells activate the synthesis of specific genes known as stress-responsive genes. The induction of these genes requires heat shock transcriptional factors (HSFs), which bind to the heat shock promoter elements and trigger the expression of stress proteins, commonly referred to as molecular chaperones or “heat shock proteins” (Hsps) [2]. Molecular chaperones play a crucial role in preventing protein misfolding, unfolding, and aggregation, thereby maintaining protein homeostasis until the stress is alleviated.

The functions of molecular chaperones depend on their co-chaperones. The co-chaperone interaction requires ATP and ions to form an active conformation, which facilitates the binding of client proteins. The 70 kDa heat shock protein (Hsp70) is the most abundant molecular chaperone among Hsps, with the highest intracellular concentration. Hsp70 exists in two main forms: the constitutive form, Hsc70, and the inducible form, Hsp72. It plays a crucial role in various cellular processes, including the folding and assembly of non-native proteins [3], the refolding of misfolded and aggregated proteins [4], the membrane translocation of organellar and secretary proteins [5], and the regulation of signaling proteins within a multiprotein Hsp90/Hsp70-based chaperone machinery [6]. Hsp70’s ability to interact with the hydrophobic segments of proteins in an ATP-dependent manner underlies these activities. Notably, the Hsp70 family exhibits significant polymorphism among human molecular chaperones [7]. Hsp70 isoforms are involved in immunity, inflammation, and stress response, and are inherited alongside MHC class I and class II genes as part of ancestral haplotypes [8,9]. Numerous studies have reported deleterious mutations of various chaperones contributing to the etiology of several diseases. For instance, Hsp70 polymorphisms have been linked to the inflammatory or autoimmune pathogenesis of conditions such as sepsis [10], Crohn’s disease [11], Alzheimer’s disease [12], pancreatitis [13], and acute graft-versus-host disease [14]. These findings underscore Hsp70’s functional diversity and its potential therapeutic implication in various human diseases.

## 2. Hsp70 Structure and Divergence

Hsp70 family members are highly conserved molecular chaperones present across all organisms. In *E. coli*, there are three members of the Hsp70 (DnaK); in *S. cerevisiae*, there are 14 members; and in humans, there are 13 members, including Hsp110, which shares structural and functional similarities with nucleotide exchange factors (NEFs) [15]. Hsp70 homologs are localized in various cellular compartments, including the cytosol, nucleus, ER, and mitochondria. Hsp70 isoforms, encoded by a multigene family, comprise 11 distinct genes in humans, eight of which have been present on five different chromosomes. These genes are located at dispersed loci, three within the MHC class III region on chromosome 6 [16], two on chromosome 1 [17], and one each on chromosomes 5 [18], 9 [19], 11 [20], and 14 [21]. Additionally, the inducible Hsp70 isoform is encoded by a gene located on chromosome 21 [19].

**Figure 1:**
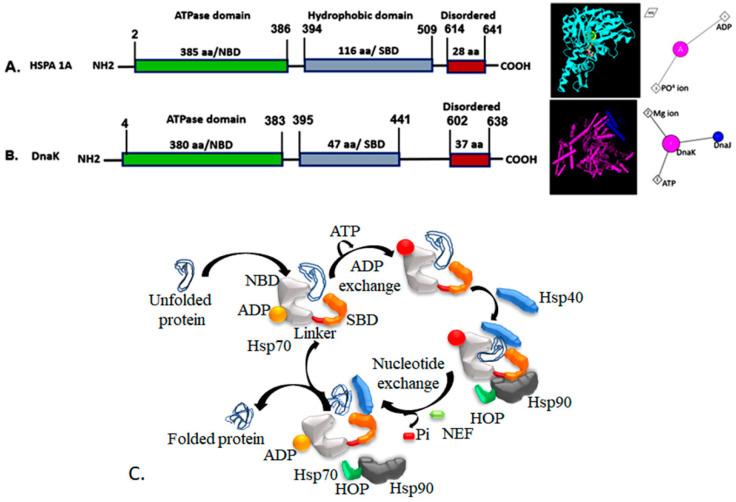
This figure illustrates the structure and function of Hsp70 chaperones. (**A**) Schematic representation of the human Hsp70 gene organization and protein structure, highlighting key domains and interacting molecules (adapted from the NCBI protein database). (**B**) Gene organization of DnaK in E. coli and the structural depiction of full-length DnaK with a bound J-domain and associated interacting partners (adapted from the NCBI protein database). (**C**) Illustration of protein folding facilitated by the Hsp70 chaperone system, emphasizing co-chaperone interactions and the ATP-dependent folding cycle.

Hsp70 family proteins exhibit conserved domains with variable amino acid sequences. Hsp70 consists of an N-terminal domain, which includes a 45 kDa nucleotide-binding domain (NBD), followed by a substrate-binding domain (SBD) composed of 10 kDa helical lid domain (SBDα), a 15 kDa β-domain (SBDβ), and a disordered C-terminal short amino acids sequence with conserved charged motifs (EEVD) that interact with specific co-factors [22,23] (Figure 1A,B). In eukaryotes, the peptide-binding domain of Hsp70 comprises two structural units: an eight-stranded antiparallel β-sandwich with a hydrophobic groove on its upper surface and a helical α-domain positioned on top [24]. The β-sheets are interconnected by loops, forming a hydrophobic binding pocket (~5 × 7 Å), in which the leucine residue of bound peptides is buried [25].

The ATPase domain of Hsp70 consists of two large globular subdomains, I and II, separated by a cleft and further divided into four sub-domains: IA, IB, IIA, and IIB [3]. Nucleotides bind at the base of this cleft, coordinated by one Mg^+2^ and two K^+^ ions, interacting with all four subdomains [26]. These interactions stabilize the ADP-bound conformation of the NBD. Nucleotide exchange factors (NEFs) are well-established co-chaperones that regulate the ATP-ADP cycling of Hsp70. In humans, Hsp70s utilize at least 50 different J- proteins and eight different NEFs. The ATP-bound conformation of Hsp70 has a low affinity for substrates, leading to their dissociation [27]. ATP hydrolysis converts Hsp70 from a “fast binding, fast release” state to a “slow binding, slow release” state, stabilizing substrate interactions, and acting as a rate-limiting step [28]. The binding of a J-domain protein (JDP/Hsp40) induces ATP hydrolysis, shifting Hsp70 into its ADP-bound state, thereby allowing its recycling for subsequent reactions (Figure 1C).

Peptide library screening for substrates of DnaK and the ER-residing Hsp70, BIP, revealed that these substrates predominantly contain hydrophobic residues within the substrate-binding cavity and positively charged residues outside it [29,30]. Bag-1, a heterogeneous family of multidomain proteins, plays a crucial in stimulating nucleotide exchange in mammalian homologs of Hsc70 and Hsp70 [31]. It interacts with Hsc70, which destabilizes the ADP-bound state, facilitating subsequent ATP hydrolysis cycles [31]. The substrates for Hsp70 vary widely in sequence and structure, ranging from completely unfolded nascent polypeptides emerging at ribosomes and translocation pores [32] to negative regulatory proteins, such as clathrin [33], transcription factors like HSF, and steroid hormone receptors [34]; kinases, including Raf and CIF2-kinase [35]; and p53 and DNA replication proteins, including λP, RepE, and RepA [36]. This extensive functional versatility underscores the critical role of Hsp70 in protein folding, stabilization, and cellular stress responses, making it a central player in maintaining cellular integrity under both physiological and stress conditions.

### 2.1. Nuclear Localization and Functions of Hsp70

The heat shock response is characterized by both the induction of Hsps and their translocation to cellular organelles and the nucleus [37,38,39]. The Hsp70 is predominantly localized in the cytoplasm under normal conditions; however, it accumulates in the nucleus upon stress, including heat, hypoxia, hepatic inflammation, cardiac ischemia, and ROS [40]. Small molecules less than 40 kDa can passively diffuse into the nucleus, whereas proteins above 40 kDa cannot pass through the nuclear pore complex (NPC) alone [41]. To pass through the nuclear pore complex (NPC), they require carrier proteins collectively referred to as nuclear transport receptors (NTRs), such as importins-β [42]. Hikeshi, a specific type of NTR, facilitates the nuclear import of Hsp70 [43]. Furthermore, mutations in the Hikeshi gene, such as p.Val54Leu and p.Cys4Ser, have been shown to inhibit Hsp70’s nuclear import under heat stress in fibroblast cells, underscoring its vital role in this process [44]. Additionally, the nuclear transport of Hsp70 is facilitated by a nuclear localization sequence (NLS), a short motif of 4–15 amino acids enriched in the basic residues such as lysine and arginine, often flanked by helix-breaking residues like proline and glycine [45]. Site-directed mutagenesis has identified three possible NLSs in human Hsp72: NLS1 (amino acids 245 to 264; KRKHKKDISENKRAVRRLR), NLS2 (amino acids 568–574, KKKYLDK), and NLS3 (amino acids 595–598; HKRK) [39,46]. Mutational studies involving alanine substitution of lysine and arginine to preserve secondary structure revealed that deletion of amino acids 245–250 (KRKHKK) reduced nuclear accumulation but did not completely abolish it [47]. A functional nuclear export signal (NES) has been identified in the C-terminal domain of *S. cerevisiae* Ssb1p, a homolog of human Hsc70, however, the corresponding region is absent in human Hsc70, leaving the molecular mechanism for its nuclear export unexplored. Notably, a novel variant of human Hsp70, Hsc54, contains a functional leucine-rich NES (^394^ LDVTPLSL^401^) but lacks the NLRS (473–492), a 20-amino acid sequence crucial for nuclear retention of Hsc70. This difference facilitates the cytoplasm export of Hsc54, whereas the NLS is essential for the prolonged retention of Hsc70 in the nucleus [48]. Hu et al. found that Hsp70 associates with nuclear speckles in a manner dependent on Hsp70 promoter sequences. Further investigation is required to confirm the association of nascent transcript with nuclear speckles [49].

Recent studies highlighted that post-translational modifications (PTMs), including phosphorylation and O-linked N-acetylglucosamine (O-GlcNAc) modification, play crucial roles in nucleocytoplasmic forms of Hsp70 lectin activity and facilitating NLS-independent nuclear transport [50]. Another study suggests that N-acetyl-D-glucosamine (GlcNAc)-specific lectins derived from adult rat liver cells may act as shuttling molecules aiding in nucleocytoplasmic transport of Hsp70 [51,52]. While the molecular chaperone function of Hsp70 is well studied, its nuclear functions remain less understood. In humans, Hsp70 has been demonstrated to play a role in erythroid differentiation by binding to GATA-1, preventing its degradation by caspases, and thereby supporting erythroid maturation [53]. Furthermore, nuclear Hsp70 suppresses lipopolysaccharide-induced inflammation in dendritic cells by promoting the degradation of the p65 subunit of Nf-kb [54]. Additionally, its interaction with VHR phosphatase attenuates neuronal cell death caused by glutamate excitotoxicity [55]. Another variant, GRP78, has been implicated as a transcriptional modulator in cancer [56]. In human lung cancer, nuclear localization of GRP78 leads to the sequestration of inhibitor of DNA binding 2 (ID2), a transcriptional factor that regulates EGFR protein expression [57]. Further research is needed to explore the diverse nuclear functions of Hsp70 and its association with the ribosomal protein complex, which could have therapeutic applications in various diseases.

### 2.2. Hsp70 Assist Protein Folding and Regulate Hyperthermia

The Hsp70 family of proteins assists in protein folding through its ATP-binding capability, enabling regulation via cycles of co-chaperone binding and release until the substrate achieves its native conformation [58]. In yeast and S. cerevisiae, cytosolic Hsp70 homologs specifically bind to nascent proteins, whereas small proteins often fold independently after synthesis and release [32,59]. Hsp70 proteins, in conjugation with co-chaperones such as DnaJ homologs and Hsp100 homologs, facilitate protein folding and promote post-translational modifications of nascent proteins [3]. During translation, the exposure of hydrophobic side chains in nascent proteins increases the risk of aggregation, especially in the crowded cytosolic environment [60]. Moreover, Hsp70s can actively modulate protein structure through their secondary amid peptide bond cis-trans isomerase (APIases) activity, which selectively accelerates the cis-trans isomerization of non-prolyl peptide bonds, thereby promoting proper folding [61].

In eukaryotes, cellular organelle-specific Hsp70 homologs perform essential functions. The ER luminal Hsp70, Bip/Kar2P in yeast [62], and mitochondrial matrix Hsp70 (mtHsp70) [63] assist in the translocation of preproteins through cis-trans conformational changes driven by trapping and pulling mechanisms. The stress-inducible Hsp70, along with its cognate form Hsc70 and the distantly related Hsp110 family members, exhibit RNA-binding properties [64]. Hsp70 functions as an RNA-chaperone, facilitating RNA folding and exposing critical motifs essential for regulatory events during translation and/or decay. Furthermore, Hsp70 family chaperones, along with their co-chaperones, interact with a diverse array of signaling molecules, including nuclear hormone receptors [65], tyrosine and serine/threonine kinases [35], and regulators of the cell cycle and apoptosis [66]. HspA5, an isoform of Hsp70, mainly localizes to the ER, where it regulates protein folding during ER-associated stress [67], but cellular stimuli such as ER stress and ER-associated degradation can trigger its localization to the mitochondria and cytosol [68]. In humans, HspA5 is upregulated in prefrontal cortex neurons of ALS patients, and its increased cytoplasmic expression appears to mitigate TDP-43-induced toxicity in *Drosophila*, identifying HspA5 as a potential target in TDP43-associated diseases [69].

## 3. Hsp70: A Key Regulator in Human Diseases

Hsp70 functions have been extensively studied in various pathological conditions and are implicated in several diseases, including inflammatory disorders, neurodegenerative diseases such as Alzheimer’s disease, cardiovascular disease, diabetes, bone disorders, and multiple cancers [70]. In humans, Hsp70 consists of multiple isoforms that exhibit differential expression in response to stress stimuli. However, the specific role of individual isoforms, such as HspA1A, HspA1B, HspA1L, HspA2, and HspA5, in various disease conditions, remains largely uncertain. Recent studies have identified specialized heterocomplex chaperone assemblies, known as epichaperomes-dynamic scaffolding platforms that regulate protein–protein interaction networks. By stabilizing these interactions, epichaperomes influence cellular stress responses and contribute to the persistence of pathological states in diseases such as cancer and Alzheimer’s disease [71]. Post-translational modifications (PTMs) of molecular chaperones create a microenvironment conducive to epichaperome formation, further promoting disease progression [72]. While several studies have focused on Hsp90 and Hsp70 epichaperome assemblies, the precise role of Hsp70 and its co-chaperones in many diseases remains unclear. The current review highlights the diverse functions of Hsp70 in human diseases and its impact on disease pathology.

### 3.1. Hsp70 in Various Cancer

Hsp70 expression is induced in many cancers, where it promotes tumorigenesis and is associated with poor survival [73]. Various Hsp70 isoforms play critical roles in cancer progression and survival. In cancer, elevated expression of Hsp70 can lead to its translocation into extracellular vesicles released by tumor cells, triggering pro- or anti-tumorigenic responses [74]. However, prolonged exposure to eHsp70 can desensitize cancer cells to immune responses, facilitating long-term survival and promoting tumor growth [75]. Consequently, Hsp70 has also been hypothesized as a potential adjuvant in cancer immunotherapy. For instance, in vitro knockdown of Hsp70 in gastric cancer cells has been shown to inhibit cell proliferation, induce cell cycle arrest, and increase apoptosis [76]. Elevated serum Hsp70 levels have been observed in patients with acute lymphocytic leukemia (ALL) [77], colorectal cancer [78], gastric cancer [79], pancreatic cancer [80], and breast cancer [81]. Elevated expression of Hsp70 suppresses apoptosis by inhibiting key regulators such as apoptosis signal-regulating kinase 1 (ASK1) [82], C-jun terminal kinases (JNK) [83], Bcl-2 associated X apoptosis regulator (BAX) [84], apoptosis protease activating factor 1 (APAF-1) [85], and apoptosis-inducing factor (AIF) [86]. Furthermore, in various cancers, Hsp70 inactivates p53, thereby promoting tumor progression [87]. In contrast, lung cancer patients exhibit significantly reduced plasma Hsp70 levels compared to healthy controls [88]. In non-small cell lung cancer (NSCLC) cells, Hsp70 regulates autophagy by negatively modulating AMPK signaling. The combined inhibition of Hsp70 and autophagy has been shown to synergistically reduce tumor cell metabolic activity, growth, and viability in NSCLC cells [89].

**Figure 2:**
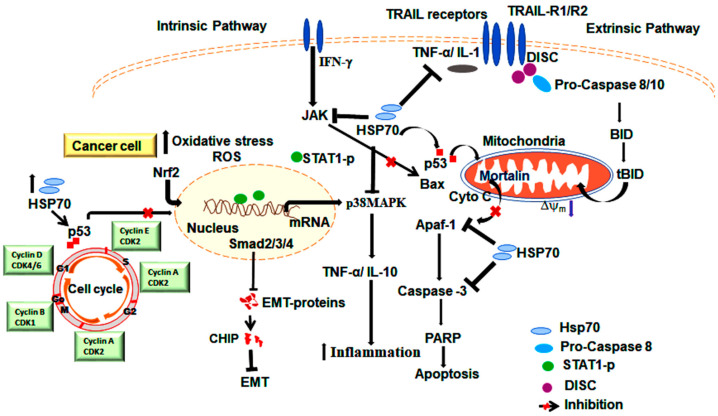
This figure illustrates the role of Hsp70 in various cellular processes. The image shows different signaling pathways involved in cancer. In cancer, Hsp70 interacts with different regulatory proteins and downstream signaling cascades implicated in growth and survival.

Elevated Hsp70 levels are strongly associated with cancer metastasis, such as lymph node metastasis in breast cancer [90]. In MCF-7 breast cancer cells, Hsp70 levels are upregulated, providing protection against hyperthermia and promoting proliferation by shortening the G0/G1 and S phases of the cell cycle [91]. Hsp70 also prevents anoikis, a form of cell death that occurs when cells detach from the extracellular matrix (ECM). Hsp70 stabilizes survival pathways in detached cancer cells, and enables their survival in the bloodstream or lymphatic system, facilitating distant metastasis [92]. Conversely, Hsp70 can stabilize key cell adhesion protein complexes like E-cadherin-catenin, thereby inhibiting cancer cell migration. However, downregulation of Hsp70 leads to the destabilization of the E-calmodulin-linked protein complex, resulting in enhanced tumor cell migration [93]. In human liver cancer (Huh-7 cells), Hsp70-peptide complexes (Hsp70-PC) promote EMT by activating pathways such as p38 MAPK, thereby enhancing the metastatic potential of cancer cells [94]. Additionally, Hsp70 attenuates TGF-β-mediated EMT signaling by interfering with the phosphorylation of proteins such as Smad2, Smad3, and Smad4 (Figure 2) [95]. The co-chaperone CHIP also contributes to EMT inhibition by facilitating the degradation of EMT-promoting proteins [96].

HspA9, an isoform of Hsp70, also known as Mortalin, is primarily localized in mitochondria and plays essential roles in both mitochondrial and extra-mitochondrial functions. Mortalin has been implicated in various cancers, including gastric, liver, and breast cancers [97,98,99]. It binds to p53 and sequesters it in the cytoplasm [100], and activates the expression of genes responsible for tumor suppression [101]. The interaction of HspA9 and p53 leads to the inactivation of p53’s tumor suppressor activity [102]. This phenomenon has been observed in vitro in multiple cancer cell lines, including NIH/3T3, A-172, U-2 OS, and HeLa, where its interaction with HspA9 prevents nuclear translocation, thereby inhibiting its transcriptional activity and promoting its degradation via the proteasome [103]. In contrast, this interaction has not been observed in normal cells such as MEF and TIG-3 cells [102]. As a result, HspA9 enables cancer cells to evade apoptosis by disrupting p53’s tumor-suppressor functions, thereby enhancing cell survival. In pancreatic ductal adenocarcinoma (PDAC) cells, inhibition of Hsp70 induces mitochondrial swelling, leading to mitochondrial apoptosis. In vivo, Hsp70 inhibition promotes AMPK-mediated autophagy flux, thus inhibiting PDAC growth [104].

Another isoform, HspA8 (Hcc70/Hsp73/Hsp71;73 kDa;646 aa), is located in the cytoplasm, nucleus, extracellular exosomes, and at the cell membrane, primarily functioning in the cytoplasm. HspA8 is a crucial molecular regulator of chaperone-mediated autophagy, acting as a detector of substrates processed by this specialized autophagy pathway [105]. It contributes to cancer cell proliferation by assisting in the folding of Cyclin D1 and supporting the formation of the Cyclin D1/CDK4 holoenzyme complex, which triggers cancer cell proliferation [106]. Histone chaperone networks, including Hsc70 and chromatin assembly factor 1(CAF1), have been shown to guide H3.1 positioning and function [107]. For this function, p53 appears to down-regulate nuclear phosphatidic acid (PA) levels, likely via its transcriptional activity, and exclude EZH2 from the H3.1 interactome [108]. HspA8 is highly overexpressed in endometrial carcinoma tissues. The depletion of HspA8 siRNA in RL-95-2 and HEC-1B cells suppresses cell proliferation by reducing the G0/G1 phase percentage and increasing the S-phase percentage [109]. These findings indicate that targeting HspA8 inhibition or disrupting its interactions with other proteins could help suppress tumor growth.

Hsp70 has been shown to elicit cytotoxic T lymphocyte (CTL) responses against peptides bound to it in various cancers. In vivo immunization with clones derived from a primary tumor did not induce Hsp70 activation [110]. However, when the tumor cells were transfected with a gene encoding the Hsp70 hybrid complex, they became susceptible to T cell-mediated lysis. This suggests that inducible Hsp70, following heat stress, enhances immunogenicity and provides signals to antigen-presenting cells for priming CD8+ T cells [111,112]. These findings confirm that inducible Hsp70 becomes more immunogenic after stress and that heat shocked cells stimulate both the innate and adaptive immune response [110]. Depending on its interacting proteins, Hsp70 may exhibit either pro-tumorigenic or anti-tumorigenic effects, highlighting its complex role in cancer progression and metastasis. Elevated serum levels of Hsp70 in various cancer patients suggest its potential as a biomarker for disease progression, warranting further investigation across multiple cancer types.

### 3.2. Hsp70 in Oxidative Stress and Inflammation

**Figure 3:**
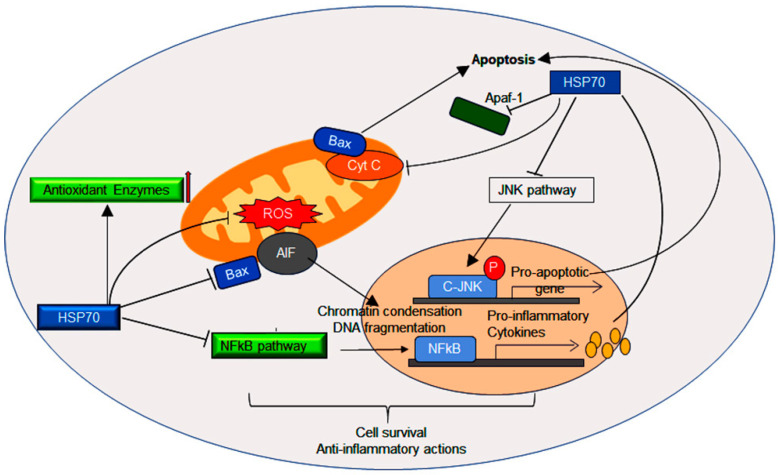
This image illustrates the regulation of oxidative stress and associated signaling pathways. It depicts the role of Hsp70 in assisting mitochondrial stress management and handling reactive oxygen species (ROS) through the JNK and NF-κb signaling pathways, along with downstream signaling molecules. Additionally, Hsp70 regulates inflammation and apoptosis by interacting with various cytokines and anti-apoptotic proteins, which are implicated in cell survival and anti-inflammatory activities.

Oxidative stress can lead to the oxidation of proteins, lipids, and DNA, potentially resulting in cytotoxicity and ultimately cell death. Cysteine (Cys) residues are particularly susceptible to oxidative modifications, facilitating redox signal transduction within various cellular pathways [113,114]. Members of the Hsp70 family are closely related to redox homeostasis and contain at least one Cys residue, which undergoes PTMs (especially cys modification) induced by oxidative stress. This oxidative stress-induced expression of Hsp70 impacts redox homeostasis [115,116]. Under conditions of increased oxidative stress, HSFs and the redox-sensitive transcription factor nuclear factor erythroid 2-related factors 2 (Nrf2) are activated through KEAP1 in response to electrophile (Figure 3) [117]. PTMs, particularly those modifying Cys residues, regulate the Hsp70 function by altering its interactions with client substrates and co-chaperones. Human HspA1A contains five Cys residues: Cys17, Cys267, and Cys306, located in NBD, and Cys574 and Cys603, in the SBDα domain. These residues undergo reversible cysteine modifications, including glutathionylation [118]. Glutathionylation of Cys574 and Cys603, located in the C-terminal α-helical lid of the SBD, inhibits the substrate-binding site, thereby promoting intrinsic ATPase activity and competing with the binding of external substrates, including Hsf1 [118]. Studies have shown that the induced expression of Hsp70 upregulates Akt-eNOS activity, leading to an increase in NO, which indirectly suppresses mitochondrial ROS (mtROS) by quenching superoxide [119]. Furthermore, overexpression of Hsp70 (HspA1A) provides protection against the Pneumolysin (PLY)-induced mtROS and cell death in human pulmonary microvascular endothelial cells [120], highlighting Hsp70 as a promising therapeutic target in streptococcus-induced pneumonia.

The immunoreactivity of Hsp70 varies depending on its localization. iHsp70 exhibits anti-inflammatory properties, while eHsp70 can induce both anti-inflammatory and pro-inflammatory responses [30]. The induction of Hsp70 prevents interleukin-6 (IL-6) and nitric oxide (NO) production in serum, as well as apoptosis and tissue damage in the intestinal tract in vivo. This finding suggests that Hsp70 protects cells against TNF-induced lethality following heat shock [121].

Hsp70 suppresses the expression of NO synthase, which is directly linked to NF-κB activity. The binding of Hsp70 to Rel family proteins, such as p65, downregulates the NF-κB/Rel complex during the acute phase of inflammation [122]. Hsp70 stabilizes I-κB and/or prevents nuclear translocation and subsequent iNOS promoter activity [123], thereby reducing pro-inflammatory gene expression. These findings suggest that Hsp70 may confer a novel mechanism of NF-κB regulation in cells affected by high temperature or other factors.

Paradoxically, eHsp70 can exert a wide range of functions, either triggering pro-inflammatory cascade signaling or, in cases of immune system over-activation, suppressing it [124]. It is secreted by neurons, epithelial cells, embryonic cells, B lymphocytes, dendritic cells, mature erythrocytes, and tumor cells [125,126]. Cytoplasmic Hsp70 interacts with shuttle proteins to reach the plasma membrane [127] and is secreted via a non-classical secretion pathway involving lysosomal lipid rafts prior to exocytosis [128,129]. Under cellular stress, Hsp70 and its constitutive form, Hsc70, bind to phosphatidylserine regions of the cell membrane, rapidly inserting into the lipid bilayer. Once embedded, they are packaged into exosomes and released, leading to the activation of immune cells such as macrophages [127]. As a cytokine, eHsp70 induces the release of pro-inflammatory cytokines, including TNF-α, IL-6, and IL-1β from monocytes [124,130]. The release of Hsp70 from dying cells acts as a danger signal, whereas its secretion from living cells reflects a successful stress response [131]. The interaction of Hsp70 with membranes acts as a platform for its release into the extracellular environment, activating the immune response, particularly macrophages [132], and thereby serving as a danger signal.

### 3.3. Hsp70 in Neurodegenerative Diseases

**Figure 4:**
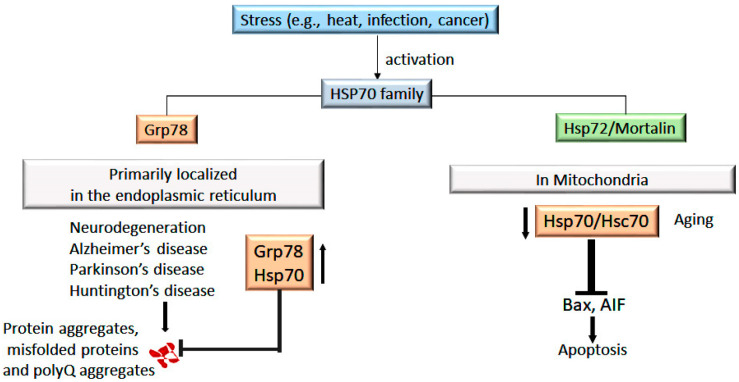
This image illustrates the role of Hsp70 in aging and apoptosis, highlighting the function of endoplasmic reticulum (ER)- and mitochondria-specific Hsp70 homologs under stress conditions. It depicts the involvement of Grp78 (ER) and Mortalin (mitochondria) in neurodegeneration, emphasizing their interactions in mitigating protein toxicity and alleviating the detrimental effects of stress-associated conditions in aging and neurodegenerative diseases.

Hsp70 is expressed in various types of neurons and plays a crucial role in protecting them from damage under pathological conditions such as traumatic nerve injury, stress, excitotoxicity, stroke, and oxidative stress [133,134]. A progressive decline in proteostasis mechanisms, including impaired proteasomal activity and reduced efficacy of molecular chaperones, exacerbates protein aggregation, contributing to neurodegeneration [135]. Hsp70 is crucial in sequestering β-amyloid and other misfolded proteins, thereby mitigating their toxic effects [136]. For instance, in Alzheimer’s disease (AD), an age-related cognitive disorder, the induction of Hsp70 has been observed in affected neurons and neighboring astrocytes. The amyloid precursor protein (APP), an integral plasma membrane protein processed in the ER, depends on the ER-resident Hsp70 homolog Grp78 for refolding and neuronal survival [136,137]. Additionally, Hsp70 inhibits α-synuclein fibrillization in an ATP-dependent manner, acting as a “holdase” to stabilize intermediates and prevent pathological aggregation. Notably, α-synuclein interacts with the SBD of Hsp70 through a canonical interaction, while recent evidence suggests additional yet poorly characterized, noncanonical binding sites on Hsp70 [138]. In AD, the formation of protein aggregates exacerbates synaptic abnormalities and memory impairment, particularly with aging. A critical factor contributing to this process is the shift from the chaperome to epichaperomes, which disrupts protein–protein interactions, leading to synaptic dysfunction and cognitive decline, as observed in PS19 mice. Notably, inhibition of Hsp90-Hsp70 epichaperome complex using small-molecule inhibitors such as PU-H71 and PU-AD has been shown to restore synaptic integrity and cognitive performance [139]. These interactions represent promising therapeutic targets for modulating protein aggregation in neurodegenerative diseases. Similarly, in Parkinson’s disease (PD)—a progressive neurodegenerative disorder characterized by the loss of midbrain dopaminergic neuron (mDA)—interventions such as dietary restriction and 2-deoxyglucose administration have been shown to upregulate Hsp70 and Grp78, providing significant neuroprotection in PD models (Figure 4) [140]. Genetic stressors such as STAT3 and NF-κB enhance the phosphorylation of tyrosine hydroxylase (TH), thereby stabilizing the Hsp90–chaperome network. Inhibition of this stabilized Hsp90–chaperome network using PU-H71 compounds has been shown to promote axonal growth in in vivo PD models [141]. Overall, targeting the Hsp90-Hsp70 chaperone complex with inhibitors like PU-H71 and PU-AD presents a promising therapeutic strategy for both AD and PD.

Hsp70 also plays a vital role in managing protein aggregates in polyglutamine (polyQ) expansion disorders, such as Huntington’s disease (HD), Kennedy’s spinal and bulbar muscular atrophy, spinocerebellar ataxias, and Machado–Joseph disease [142,143] Elevated levels of Hsp70 and Hsc70 effectively inhibit polyQ protein aggregation and delay disease progression [144]. In vivo, the administration of exogenous Hsp70 (eHsp70) has been shown to mitigate nerve injury in a model of sciatic nerve transection in rats [145]. The eHsp70 stabilizes PINK1, preventing its degradation and enhancing anti-inflammatory effects in dorsal root ganglion (DRG), thereby promoting DRG neuronal survival and axon regeneration [146]. However, the underlying mechanism remains incompletely understood. Additionally, Hsp40, a co-chaperone of Hsp70, counteracts prion protein aggregation, which is implicated in Creutzfeldt–Jakob disease [147]. Native prion proteins, typically adopting an α-helical conformation, contribute to essential cellular functions such as signaling, copper metabolism, redox regulation, and neuronal protection [148]. However, their misfolding into β-sheet-rich conformations renders them protease-resistant, leading to excessive aggregation and neurotoxicity [149]. Grp78 facilitates the degradation of these misfolded prion proteins through the proteasomal pathway, underscoring its critical role in maintaining proteostasis [150].

### 3.4. Hsp70 in Cardiovascular Diseases and Atherosclerosis

Hsp70 plays a crucial role in maintaining cardiac integrity under stress conditions [151]. Its induction can prevent cardiac muscle damage caused by both ischemia and reperfusion [152]. An analysis of 222 cardiovascular disease patients revealed a positive correlation between Hsp70 level and heart failure (HF). Elevated Hsp70 levels activate CD14/Toll-like receptor-4 and induce the expression of inflammatory cytokines, which in turn promote Hsp synthesis [153]. The source of circulating Hsp70 in congestive heart failure (CHF) remains controversial; potential origins include white blood cells via CD14 receptor activation [154], the myocardium itself [155], or the endothelium [156]. Notably, Hsp70 levels progressively increase with advancing stages of HF (*p* < 0.0001), and its concentration is significantly elevated in stage B HF patients compared to stage A. This suggests that the enhanced levels of Hsp70 serve as an innate protective mechanism, aiding in the restoration of physiological conditions [153]. Although, NT-proBNP (N-terminal pro-B-type natriuretic peptide) is widely used as a diagnostic and prognostic biomarker in chronic heart failure [157], receiver-operating curve (ROC) analysis in stage B HF patients has demonstrated that Hsp70 is more sensitive than NT-proBNP. This finding underscores the potential of Hsp70 as an early biomarker for heart failure progression [153].

Hsp70 can be induced by ischemia, nutrient deprivation, irradiation, infections, and inflammation. Studies in mice have demonstrated that eHsp70 plays a protective role in response to hypertension [158]. This highlights Hsp70’s involvement in the entire pathophysiological progression of HF, as well as its potential role in organ transplantation, where pretreatment with Hsp70 enhances organ survival [159]. Furthermore, transfection of the Hsp70 into rat hearts has been shown to protect the ischemic myocardium [160]. For instance, the combination of non-steroidal anti-inflammatory drugs (NSAIDs), such as sodium salicylate, with mild heat shock, activates HSF without inducing the Hsp72 response. However, exposure to severe heat shock (42 °C) significantly elevates myocardial Hsp72 levels, improving post-ischemic recovery, as well as contraction and relaxation rates [161]. In pediatric patients undergoing surgery for congenital heart defect (CHD), ischemic stress does not significantly affect Hsp70-1 mRNA expression, likely due to the high basal levels of Hsp70 protein [162]. In atherosclerotic plaques, various cell types, including macrophages and dendritic cells (DCs), overexpress Hsp70. The induced Hsp70 in DCs may activate T cells expressing CD1d, which stimulates lipid antigen-presenting cells to secrete inflammatory cytokines within atherosclerotic lesions [163]. This indicates that Hsp70 overexpression by DCs might contribute to the early stages of atherogenesis. Elevated Hsp70 levels in plasma may also serve as a biomarker for HF and atrial fibrillation [151]. Conversely, Geranyl-geranyl-acetone (GGA) has been shown to induce Hsp70 and Hsp72 mRNA expression in ischemia–reperfusion in rat liver [164]. While iHsp70 inhibition has been shown to reduce myocyte fibrosis, blocking Ec-Hsp70 with anti-Hsp70 antibodies has been shown to mitigate hypertension-induced cardiac hypertrophy and fibrosis [158]. These findings highlight the dual role of HSPs in cardiac pathology, emphasizing the need for targeted therapeutic strategies based on disease context.

### 3.5. Hsp70 in Aging and Apoptosis

Aging and apoptosis are closely associated with tissue deterioration. Aging is characterized by an increased rate of protein modification, which exacerbates folding homeostasis. Consequently, the impairment of chaperone functions results in protein misfolding and aggregation. However, the activity of the major cytoplasmic proteolytic apparatus, the proteasome, also declines with aging and is further compromised by glycation [165]. In vivo studies on aging rats have shown no significant increase in heat-induced Hsp70 levels with heating, but a large increase with exercise [166]. These data suggest that the blunted HSP response to heating with aging is not due to an inability to produce inducible Hsp70, but rather that aged rats have a robust response to exertional hyperthermia. Additionally, Hsp70 induction is impaired in fibroblasts and hepatocytes, while skeletal muscles in aged rats displayed increased Hsp70 levels, which affect its nuclear export [167]. This impairment in post-transcriptional processing and nuclear export leads to reduced functional Hsp70 levels, ultimately resulting in cell death [168]. Aged organisms exhibit failure to induce Hsp72 and other Hsps in response to stresses, which correlates with the increase in morbidity and mortality under severe stress conditions [169]. This hypothesis is supported by primary human fibroblast cells (IMR90), which failed to induce Hsp72 after pretreatment with mild heat shock. When subjected to severe heat shock, these cells activate JNK, resulting in apoptosis [170]. In contrast, young cells induce Hsp72, suppress JNK, and subsequently prevent cell death. In elderly mice, induction of Hsp70 by inhibiting the integrated stress response (ISR) improved testosterone synthesis [171]. Notably, Hsp70 is associated with low sperm quality and plays an anti-apoptotic role in somatic cells. For instance, inhibition of Hsp70 by YM-1, an allosteric Hsp70 inhibitor, could maintain sperm quality and function during cryopreservation at 17 °C [172].

Conversely, the general decline in immune response among elderly individuals may impair the anti-chaperone immune response, as evidenced by the reduced levels of anti-Hsp70 antibodies observed in cases of dilated cardiomyopathy [173]. Despite numerous reports highlighting the protective effects of Hsps, the mechanisms by which they may negatively influence apoptosis remain unclear. A unifying feature of these observations is the inhibition of caspase proteolytic maturation and/or activity, preventing the cleavage of their target substrates [174,175]. For instance, stress-inducible Hsp70 prevents Bid cleavage and activation in response to TNF-α, acting independently of its chaperoning ability [176]. This activity suppresses the activation of the MAP kinase–JNK pathway, thereby inhibiting the mitochondrial release of cytochrome c, Smac [177], and apoptosis-inducing factors (AIF) [178] (Figure 2 and Figure 3). Both Hsp70 and Hsc70 directly prevent the translocation of AIF from the mitochondria [179]. Recent studies suggest that Hsp70, along with Hsp40 (Hdj-1) or HsdJ (Hdj-2), plays a role in blocking NO-induced apoptosis by preventing Bax translocation to mitochondria [180]. The chaperoning activity of Hsp70 is essential for suppressing caspase activation at a point downstream of cytochrome c release but upstream of caspase-3 activation [181]. Hsp70 can inhibit the formation of a functional apoptosome by directly interacting with Apaf-1, preventing the recruitment and activation of initiator caspases such as pro-caspase-9 [86]. This inhibition may occur either by preventing Apaf-1 oligomerization or by maintaining the oligomer in a confirmation that is incompatible with pro-caspase-9 recruitment, hence blocking the exposure of the Apaf-1 CARD domain [85]. Intrinsic apoptosis is triggered by stress stimuli and cellular damage, and can be suppressed by enhanced Hsp70 expression. Hsp70 achieves this by inhibiting apoptotic pathways such as JNK and p38MAPK through complex formation with CHIP, leading to the degradation of ASK1 [82].

Extrinsic apoptosis, initiated by extracellular signals such as TNF-α or TRAIL, is inhibited by Hsp70 interactions with TRAIL receptors (TRAIL-R1 and TRAIL-R2), preventing the formation of death-inducing signaling complex (DISC), a critical protein complex required to initiate the apoptotic process [182]. Furthermore, Hsp70 reduces the activity of caspase-8, an enzyme essential for cleaving Bid, a protein critical for amplifying death signals [176]. In cancer cells, Hsp70 is also localized in lysosomes, where it stabilizes the lysosomal membrane and prevents lysis, thereby protecting these cells from lysosomal-mediated apoptosis [96]. However, other studies contradict the idea that Hsp70 expression universally protects cells from apoptosis. Some findings suggest that Hsp70 increases cellular susceptibility to death-inducing effects when TNF is combined with cycloheximide and Fas/TCR/CD3 ligation [183]. The possible mechanisms by which Hsps may inhibit TNF-induced apoptosis include the suppression of phospholipase A2 activation [184], inhibition of reactive oxygen species, and an increase in glutathione levels, intracellular calcium levels, and phosphatase activity [183].

Ceramide, a lipid mediator involved in apoptosis induction, enhances heat shock-induced apoptosis by suppressing the anti-apoptotic effects of Hsp70 through post-transcriptional regulation in HL-60 cells [185]. Similarly, the enhanced expression of Hsp70 and its co-chaperone Hsp40 reduces aggregate formation and apoptosis in cultured neuronal cell models of SBMA [186]. Additionally, cytosolic Hsp70 has been observed to translocate to the cell surface in mouse lymphoma EL-4 cells [187]. It is speculated that this extracellular pool of Hsp70 is adsorbed by live cells, thereby protecting them from apoptosis. The surface-expressed Hsp70 facilitates caspase-independent apoptosis in tumor cells by binding to and enabling the uptake of granzyme B, a family of serine proteases associated with perforin in activated T-lymphocytes and natural killer cells [188]. Moreover, Hsp70 inhibits apoptotic processes by blocking the JNK signaling pathway, preventing Bax activation, and inhibiting cytochrome C release, an essential step in apoptosis initiation. Additionally, Hsp70 interferes with key apoptotic proteins such as Apaf-1 and AIF, preventing their interaction with target proteins and ultimately blocking cell death [189]. In recent studies, the role of Hsp70 has been implicated in the mitochondrial apoptotic pathway. For instance, in intervertebral disc degeneration (IVDD), Hsp70 induction prevented compression-induced apoptosis of nucleus pulposus (NP) cells by inhibiting mitochondrial fission through the promotion of SIRT3 expression [190]. These findings underscore the complex role of Hsp70 in aging and apoptosis, suggesting its potential as a therapeutic target in various diseases.

## 4. Chemical Inhibitors and Modulators of Hsp70

Hsp70 activity is regulated by its NBD, SBD, and ATPase activity, all of which serve as potential targets for modulation. The functions of Hsp70 depend on the binding and hydrolysis of the ATP and ADP cycle, making it less susceptible to many inhibitors due to its dynamic conformational transitions. Hsp70 interacts with several client proteins involved in critical cellular functions such as chromatin regulation, kinetochore formation, mitochondrial protein processing, and MAPK signaling specificity. Furthermore, chaperones are highly modified by a range of PTMs that include phosphorylation, methylation, acetylation, and ubiquitination [191,192]. Recently, histone regulator (HIR) proteins have been identified as novel clients of Hsp70. An Hsp70 inhibitor, JG-98, leads to HIR degradation, suggesting that HSP70 is crucial for HIR stability. Similarly, Pim1/Lonp-1 (involved in mitochondrial proteostasis), Mtw1/Mis12 (essential for chromosome segregation), and Ste11 (associated with pheromone and osmotic stress response) have been identified as novel client proteins of Hsp70 [193]. Several pharmacological modulators can influence the interaction of Hsp70 with its client proteins and co-chaperones. There are six specific sites that are localized for inhibitors—four binding sites in the NBD and two in the SBD. The affinity of compounds may vary among different isoforms of Hsp70. For instance, Novolactone binds covalently to Glu residue, E444 in site 4 of HspA1A/B, which is highly conserved in all canonical Hsp70s except HspA9 [194]. Similarly, YK5 binds HspA1A/B and HspA8; however, its exact binding site has yet to be confirmed.

Some Hsp70 inhibitors block the Hsp70–JDP interaction. For instance, Mal3-101 prevents JDP-dependent Hsp70 ATPase activity without affecting its fundamental properties. Another compound, 115-7c, can activate the Hsp70–JDP interaction within the same pocket at site 6, and has been shown to modulate ATPase and protein-folding activities in a yeast model of polyglutamate disease [195]. The design of novel compounds often involves mimicking the conformation of known ligands or leveraging the structure of the peptide-binding domain of target proteins. Cholesterol glucoside has been shown to upregulate Hsp70 synthesis in human fibroblasts and exhibits anti-ulcer activity when administered orally in rats by activating HSF [196,197,198]. Another set of compounds, identified as antibacterial or antimicrobial peptides—such as drosocin, pyrrhocoricin, and apidaecin—comprises short proline-rich sequences (18–20 amino acids) derived from insects. These peptides specifically target bacterial proteins, with studies identifying DnaK, the bacterial homolog of Hsp70, as a primary target, making it a promising focus for drug design [199]. Modulating the expression of Hsp70 family proteins presents a promising approach for developing immunomodulatory therapies. Novel therapeutic compounds, including chaperone and co-chaperone inducers [200,201,202], have undergone clinical testing to restore immune function and address chaperone-related immune dysfunction. While numerous Hsp70 chemical inhibitors have been tested in vitro, many exhibit low binding affinity and are classified as Class I inhibitors. Thus, many Hsp70 chemical probes plays a dual role, either promoting or inhibiting chaperone functions by regulating Hsp70 co-chaperone and client protein assembly. Known Hsp70 inhibitors are discussed in the Table 1. More research is needed to determine its specificity to specific isoforms of Hsp70.

## 5. Conclusions and Future Prospective

Hsp70s are complex molecular assemblies that play a crucial role in protein homeostasis. While the functions of Hsp70 have been extensively studied, certain aspects of its structure–function relationship remain to be fully elucidated. This review explores Hsp70-related genes and proteins, highlighting their critical role in cellular protection against stress and their implication in various human diseases. The Hsp70 gene exhibits diverse expression patterns, existing in both constitutive and inducible forms under normal and stress conditions, and pathologies (Table 2). Notably, Hsp70 proteins display distinct localization, being found in the cytoplasm, nucleus, mitochondria, and ER, and can even be associated with transmembrane domains. Apart from intracellular functions, Hsp70 proteins are actively released into the extracellular space, where they serve as “danger signals” by interacting with Toll-like receptors (TLR2 and TLR4) on macrophages, glial cells, and dendritic cells. This extracellular signaling contributes to neuroprotection by inducing the expression of pro-inflammatory cytokines and inducible nitric oxide synthase (iNOS) in response to cerebral injury. The eHsp70 is detectable in blood and CSF, while iHsp70 in blood cells can also be measured, making it a potential clinical biomarker. However, concerns remain regarding the correlation between Hsp70 expression in blood and periphery tissues in both disease and healthy individuals. Many studies have used the generalized term “Hsp70” rather than distinguishing between its inducible form, Hsp72, and constitutive form, Hsc70, in clinical samples. Accurately identifying specific Hsp70 isoforms in clinical samples is crucial for developing targeted therapies and drug designs. Furthermore, the differential regulation of various Hsp70 isoforms in cancer remains poorly understood, with limited evidence available on their specific roles. Correctly identifying Hsp70 isoforms across different cancer types, could enhance diagnostic precision and facilitate the development of more effective therapeutic strategies. Modulating Hsp70 chaperone activity using various pharmacological agents with high neuroprotective potential, such as tamoxifen, melatonin, glutamine, and HSF-1, as well as compounds that enhance Hsp70 expression by targeting the thio-disulfide system (TDS)—including selenium compounds, glutaredoxin, glutoxin, and Angiolin—is of particular interest. These Hsp70 modulators have demonstrated neuroprotective effects in animal models, highlighting their potential for drug development in the treatment of acute cerebrovascular disorders [228].

Epichaperomes, long-lived chaperone assemblies identified in cancer cells and primary tumor specimens [71], dysregulate PPI networks to provide a survival advantage to cancer cells and tumor-supporting cells in the microenvironment. However, the mechanisms underlying epichaprome formation are not yet fully elucidated, though PTMs may play a crucial role in stabilizing specific chaperone conformations that facilitate epichaperome incorporation. These assemblies have been studied in various cancers, as well as AD and PD. Inda et al. found that these assemblies are formed following the induction of human Tau in postmortem AD brain samples [139]. Thus, disrupting epichaperomes has been shown to restore protein network activity to its original functional state. A few chemical probes, such as PU-H71, Pu-HZ151, and PU-AD, have been evaluated in in vivo disease models with promising results. However, specificity and dosage remain major concerns. Further, investigation into the mechanisms underlying epichaperome formation and the identification of PTMs in various cancers, AD, and PD may provide deeper insights into the development of more specific probes or disruptors for these diseases.

Additionally, exosomal secreted Hsp70 has been detected in peripheral blood and urine samples of patients with various cancers, including lung, breast, and ovarian cancer, highlighting its potential as a novel biomarker for cancer detection and prognosis. However, real-time detection of exosomal Hsp70 remains challenging due to technical limitations and the low accuracy of current detection methods. Overcoming these obstacles is crucial for advancing Hsp70-based tumor therapies. To develop effective Hsp70-targeted treatments, it is essential to identify specific regions of the protein capable of eliciting robust anti-tumor responses by activating both innate and adaptive immunity. Further research in this area could pave the way for novel therapeutic strategies for human diseases.

## Figures and Tables

**Table 1:** Hsp70 activity modulators and their functions in various human diseases.

Hsp70 Modulators	Targets	Characteristics	References
AEG3482	JNK activity inhibition	Facilitates Hsf1-dependent expression of Hsp70 and Hsp25	[203]
Apoptozole	Hsc70 and Hsp70	Inhibits ATPase activity by binding to its ATPase domain	[204]
AP-4-139B	Hsp70	Inhibits ATPase activity of Hsp70	[104,205]
DMT003096	Inhibits Tag-stimulation of Hsp70	Inhibits breast cancer proliferation	[206]
Displurigen	HspA8 and Hsc70	Inhibits ATPase activity	[207]
Eupalinolide A	Hsp70	Induces Hsp70 expression via inhibiting HSF1 and Hsp90	[208]
Hsp70/SIRT2-In-1	Hsp70 and SIRT2	Inhibits ATPase activity	[209]
2-Hexyl-4-pentynoic acid (Compound V)	Hsp70	Induces Hsp70-1a mRNA expression via HDAC inhibition	[210]
IDF-11774	Hsp70	Binds to allosteric pocket in its NDB of Hsp70	[211]
JG-98	Disrupts the Hsp70-Bag3 interaction	Exhibits anti-proliferative activity in cancers and tumor-associated macrophages	[212]
JG-231	Disrupts the Hsp70-Bag3 interaction	Inhibits tumor proliferation and induces apoptosis	[213]
JG-48	Binds NBD of Hsc70 stabilizing client protein interaction	Inhibits Bag-1 binding to Hsc70	[214]
KNK437	Inhibits the induction of Hsp70 mRNA levels	Inhibits stress-inducible expression of Hsp	[215]
MKT-077 (FJ776)	Binds to an allosteric site of NBD in Hsc70	Affects the stability of client protein and induces apoptosis	[212]
MAL3-101	Inhibits Hsp70–Hsp40 interaction	Inhibits ATPase activity by blocking Hsp40 interaction	[216]
Myricetin	Inhibits binding of DnaJ to DnaK	Allosterically influences the DnaK–DnaJ interaction	[217]
Novalactone	Inhibits Hp70 through a covalent interaction with Glu444	Disrupts interdomain interaction	[218]
116-9e	DNAJA1 inhibitor	Inhibits Tag-mediated activation of Hsp70	[219]
Pifithrin-µ, 2-Phenylethynesulfonamide (PES)	Inhibits Hsp70 via interacting with the ATPase binding domain	Inhibits cell proliferation in cancer (NSCLS)	[220]
Pet-16	Binds to an allosteric site of SBD of Hsp70	Induces apoptosis in multiple myeloma	[221]
S1g-10	Suppresses Hsp70/Bim PPI complex	Disrupts the Hsp70-Bim PPI complex, prevents tumorigenesis	[222]
VER-155008	HspA8, HspA5, and HspA1A	Inhibits the ATPase activity of Hsp70 and binds in the ATPase pocket	[223,224]
YK5	Cytosolic Hsp70 and Hsc70 through allosteric site 1	Allosteric site 1 of Hsp70	[225,226]
YL-109	C-terminus of Hsp70-interacting protein	Induces CHIP transcription via AhR signaling	[196]
YM-08	Hsp70/Sirt2 inhibitor	Inhibits the ATPase activity of Hsp70	[209]
YM-1	Block formation of ATP-bound form	Activates binding of Hsp70 to unfolded substrates	[227]

**Table 2:** Hsp70 involvement in various human diseases and their potential applications.

Molecular Chaperone	Human Diseases	Hsp70 Functions	Biomarker	Molecular Targets	References
Hsp70/DnaK family proteins	Diabetes and metabolic diseases	eHsp70 levels impact insulin sensitivity, hyperinsulinemia, and hyperglycemia	Serum IgA level and anti-Hsp70 IgA	TLR, TNF-α pathway	[229]
	Infectious diseases	Improve cellular immune response, viral replication	CFUs in spleen and blood samples	Reduced Hsp70 expression mitigates inflammatory cytokines	[230]
	Cardiovascular diseases and atherosclerosis	Enhance Hsp70 expression in heart failure	Serum Hsp70 levels	Blocking ERK1/ERK2, TGF-β pathway	[153,158,231]
	Oxidative stress and Inflammation	PTMs of Cys residue	Mitochondrial ROS and redox homeostasis	Blocking Nrf2, JNK, and NF-κb signaling	[113,114,117,118,121,132]
	Cancer	Inhibits apoptosis, promotes tumorigenesis, activates autophagy, induces immunogenicity	Enhanced serum Hsp70 levels	Blocking MAPK signaling, and TGF-β	[74,88,89,95]
	Neurodegeneration, disorders such as AD, PD, and HD	Prevents aggregation of Aβ, α-synuclein fibrillization, Poly Q, and PINK degradation	Increased Hsp70 levels in serum, blood, and CSF	Inhibiting APP, tau miRNAs, and α-synuclein	[134,136,138,232]
	Aging and apoptosis	Induces Hsp70 and Bid cleavage, suppresses MAPK-JNK pathway, and apoptosis	Serum and tissues, cytoplasmic Hsp70 levels	JNK and p38 MAPK, and TNF-α signaling	[82,83,86,181,184,188,190]

## Data Availability

No new data were created or analyzed in this study.

## Abbreviations

The following abbreviations are used in this manuscript:

AD	Alzheimer’s disease
APIases	Amid peptide bond cis-trans isomerase
APP	Amyloid precursor protein
CHD	Congenital heart defect
CLL	Chronic lymphocytic leukemia
CTL	Cytotoxic T lymphocyte
DISC	Death-inducing signaling complex
DGR	Dorsal root ganglion
ER	Endoplasmic reticulum
EMC	Extracellular matrix
eHSP	Extracellular Hsp70
HF	Heart failure
HSP70	Heat shock protein 70
HSFs	Heat shock transcriptional factors
HD	Huntington’s disease
Hsps	Heat shock proteins
iHsp70	Intracellular Hsp70
iNOS	Inducible nitric oxide synthase
IVDD	Intervertebral disc degeneration
JNK	c-Jun N-terminal kinases
MMP-9	Matrix metalloproteinase-9
mtHSP70	Mitochondrial Hsp70
NEF	Nucleotide exchange factors
NBD	Nucleotide-binding domain
NP	Nucleus pulposus
NSCLC	Non-small cell lung cancer
NSAIDs	Non-steroidal anti-inflammatory agents
PD	Parkinson’s disease
PKC	Protein kinase C
PTM	Post-translational modifications
ROS	Reactive oxygen species
SBD	substrate-binding domain
TLR	Toll-like receptors
